# Pigmented Peril: A fatal case of Primary intracranial melanoma in a paediatric patient

**DOI:** 10.1016/j.radcr.2025.03.070

**Published:** 2025-05-01

**Authors:** Yasmine Elsherif, Nouman Aziz, Waseem Nabi, Adnan Bhat, Ahmad Basharat, Fromer Nelli

**Affiliations:** a Internal Medicine Department, Zayed Military Hospital, Abu Dhabi, UAE; b Internal Medicine Department, Wyckoff Heights Medical Center, Brooklyn, New York, USA; c Internal Medicine Department, University of Florida, Gainesville, Florida, USA; d Internal Medicine Department, Marshfield Clinic Health System, Wisconsin, USA; e Hematology and Oncology Department, Wyckoff Heights Medical Center, Brooklyn, New York, USA

**Keywords:** Intracranial melanoma, Frontotemporal mass, Case report

## Abstract

Primary CNS melanoma is an extremely rare and aggressive malignancy, especially in pediatric patients. We present the case of a 12-year-old girl who initially presented with sudden-onset severe headache and neurological deficits, and imaging revealed a left frontotemporal mass, suspected to be an arteriovenous malformation. After developing spontaneous intracerebral hemorrhage, she underwent emergency craniectomy and partial resection, with pathology confirming primary CNS melanoma. Despite treatment with immunotherapy, her condition deteriorated, leading to progressive hydrocephalus and leptomeningeal metastasis, ultimately resulting in her death. This case highlights the diagnostic and therapeutic challenges of primary CNS melanoma in children, emphasizing its rapid progression, limited treatment efficacy, and the need for early recognition, multidisciplinary management, and further research for definitive treatment.

## Introduction

Malignant melanoma is predominantly reported in the skin and mucous membranes, [1] with rare occurrences in other areas such as the brain and spinal cord accounting for approximately 1% of all melanomas [2]. According to the neurogenic theory, melanocytic elements originate from the neural crest, which also gives rise to the leptomeningeal tissues and later develops into mesodermal structures, forming this tumor. Alternative theories, including mesodermal and ectodermal origins, have also been proposed to explain the source of the pigment. One hypothesis suggests the potential for melanization of benign structures, such as schwannomas, gliomas, melanocytic meningiomas, and meningeal melanocytosis [3].

The diagnosis of this extremely rare tumor requires the exclusion of metastatic skin melanoma and narrowing of differential diagnoses based on pathology reports to confirm its primary nature. Primary CNS melanoma (pCNS melanoma) is a severe and often life-threatening condition with few years of survival [4]. Skin melanoma cases demonstrate CNS metastasis rates between 40%-60%, which may rise to 80% in autopsy findings [5]. Consequently, distinguishing between primary and secondary metastases is critical, as treatment strategies and prognosis vary significantly. Published cases remain scarce, and data on pCNS melanoma are insufficient.

We present the case of a 12-year-old female diagnosed with pCNS melanoma, who originally presented with an excruciating headache that tragically and rapidly resulted in her death.

## Case presentation

**Fig. 1:**
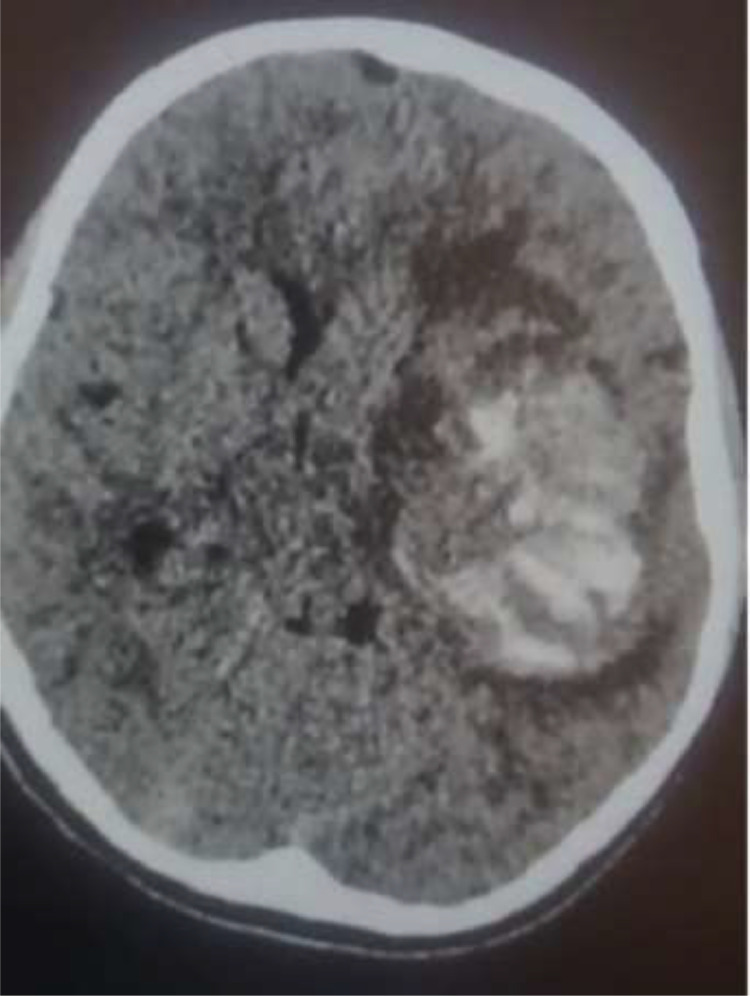
The initial CT brain imaging performed during the patient's second presentation to the emergency department. Non-enhanced CT of the brain shows a large hyperdense mass-occupying lesion in the left frontotemporal region, causing a mass effect on the left lateral ventricle. Also noted is perilesional edema with effacement of the sulci.

A 12-year-old girl with no past medical history initially presented to a secondary care facility with a sudden-onset severe headache and neurological symptoms. Computed tomography (CT) brain revealed a left frontotemporal mass near the Sylvian fissure, initially suspected to be an arteriovenous malformation (AVM). She subsequently developed a spontaneous intracerebral hemorrhage, which was managed conservatively, followed by discharge home. Shortly thereafter, she returned to the emergency department due to syncope. Repeat CT brain imaging revealed significant rebleeding, midline shift, and ventricular compression Fig. 1.

She underwent an urgent craniectomy to evacuate the hematoma and partially resect the presumed AVM. Postoperatively, her neurological status improved, with normalization of pupil size and partial recovery of right-sided hemiplegia.

**Fig. 2:**
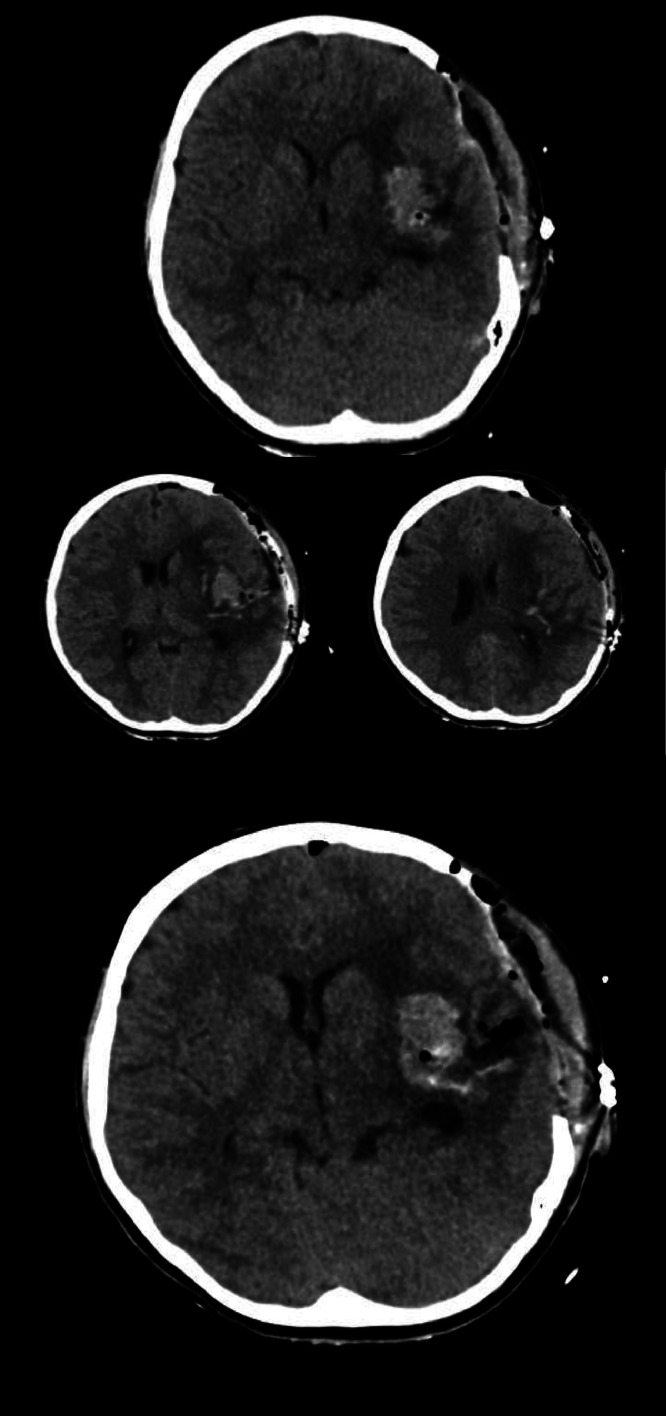
The first non-contrast CT brain following the postcraniotomy procedure. The non-contrast CT brain shows a large left parietal intraparenchymal hemorrhage with surrounding vasogenic edema, causing significant mass effect and a leftward midline shift. After the procedure, pneumocephalus is present. Further imaging, such as contrast-enhanced CT or vascular studies, may be required to assess for underlying vascular pathology.

**Fig. 3:**
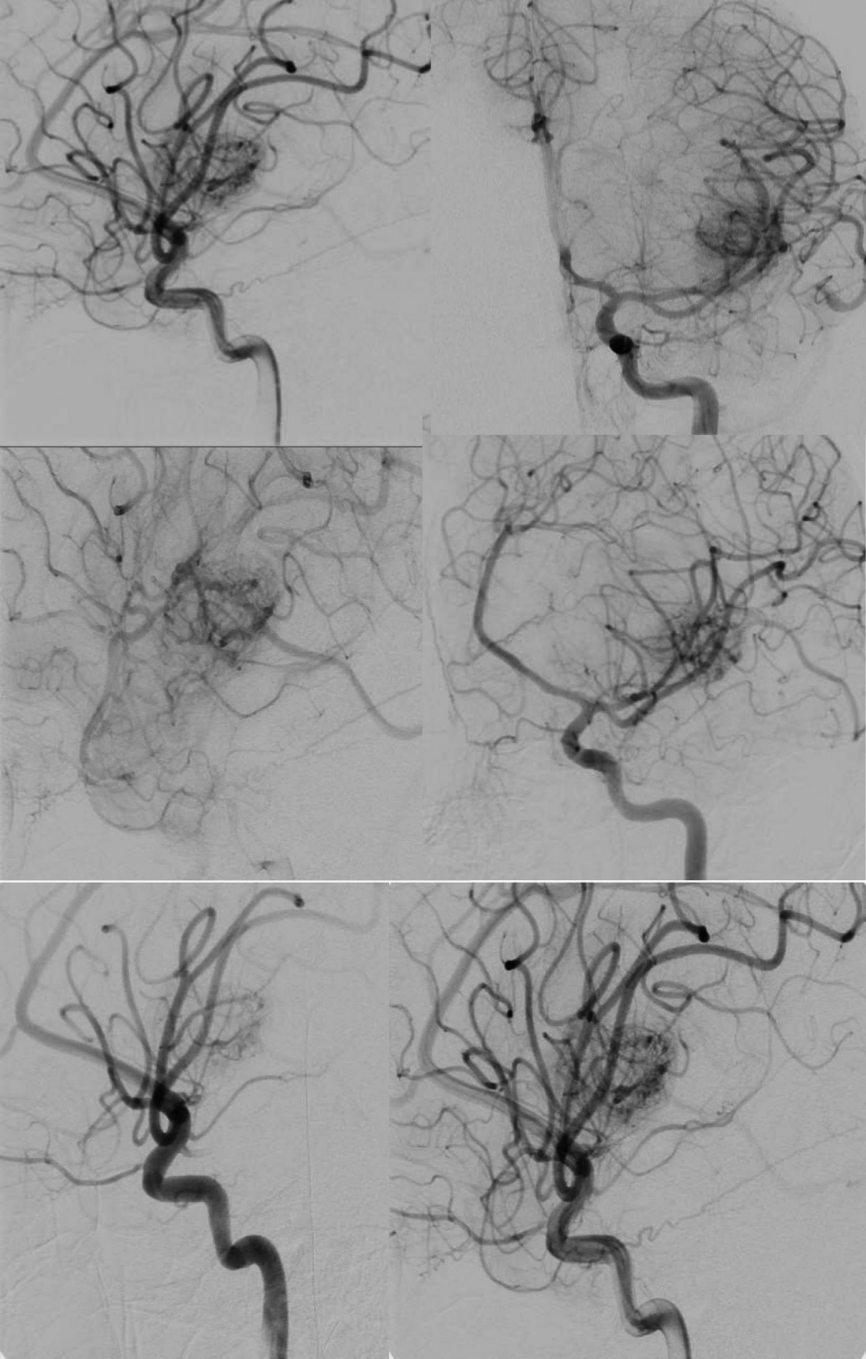
Cerebral DSA: Left insular arteriovenous malformation with high-flow shunting. Arterial cerebral digital subtraction angiography (DSA) from the left carotid artery demonstrates a highly vascular lesion in the left insular region. There is no evidence of early venous enhancement suggestive of an AVM. Additionally, no evidence of a ruptured aneurysm is seen in the images. The findings are suggestive of a high-grade AVM, and further evaluation with Spetzler-Martin grading is recommended to guide management.

**Fig. 4:**
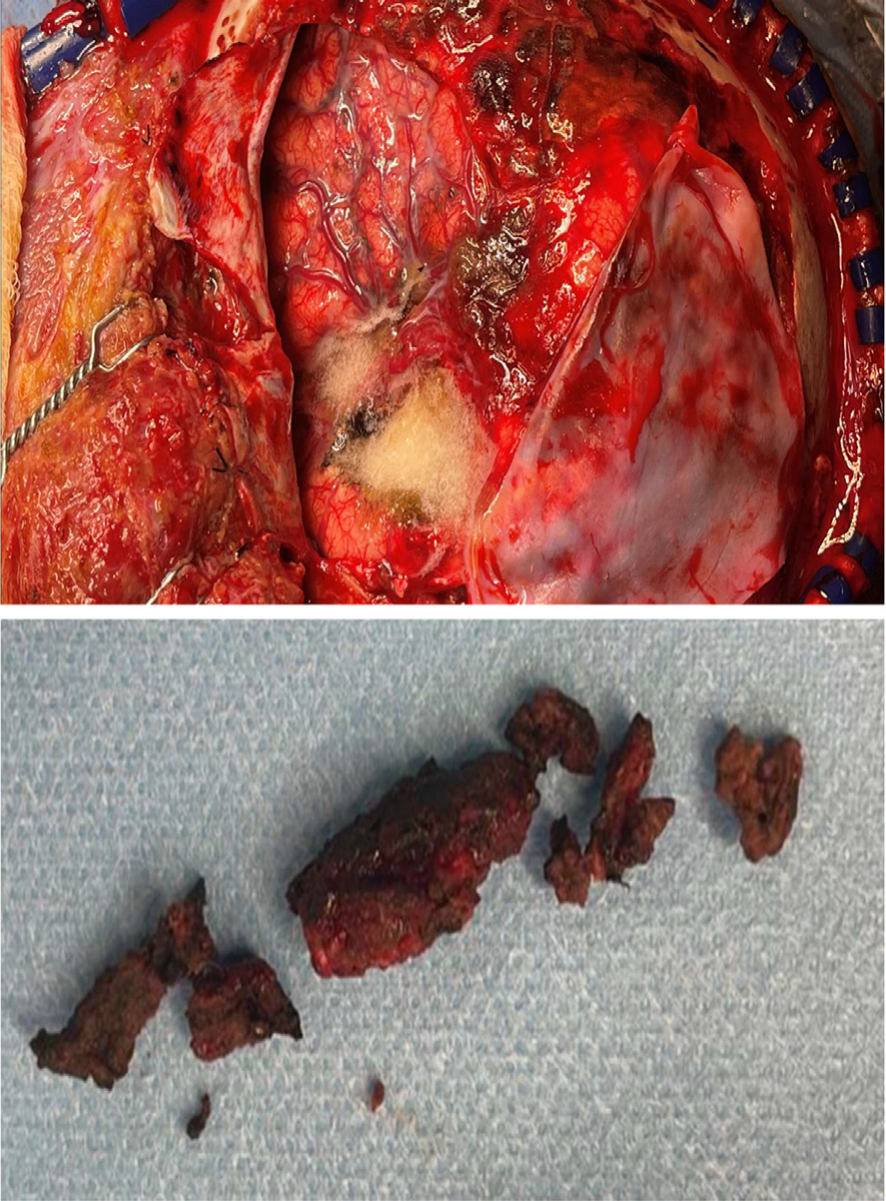
Intraoperative images.

She was thereafter transferred to our tertiary care hospital for further management. Upon admission to the pediatric ward, she was afebrile, with a pulse rate of 106 bpm, blood pressure of 82/47 mmHg, and a respiratory rate of 20 breaths per minute. Initially, she exhibited prominent aphasia and right-sided hemiplegia, which improved over time. A postoperative non-contrast head CT scan revealed intraparenchymal hemorrhage involving the left insular, lentiform, and amygdala regions. Gas locules within the left temporal lobe hematoma were presumed to be surgical artifacts Fig. 2. Angiography identified blush enhancement medial to the M2 segment of the left MCA superior division near the hemorrhage site. A second surgery was performed at our facility to achieve total gross resection of the mass Fig. 3. During the procedure, most of the tumor was resected, except for a residual blush close to the main MCA trunk and M3 branch Fig. 4.

**Fig. 5:**
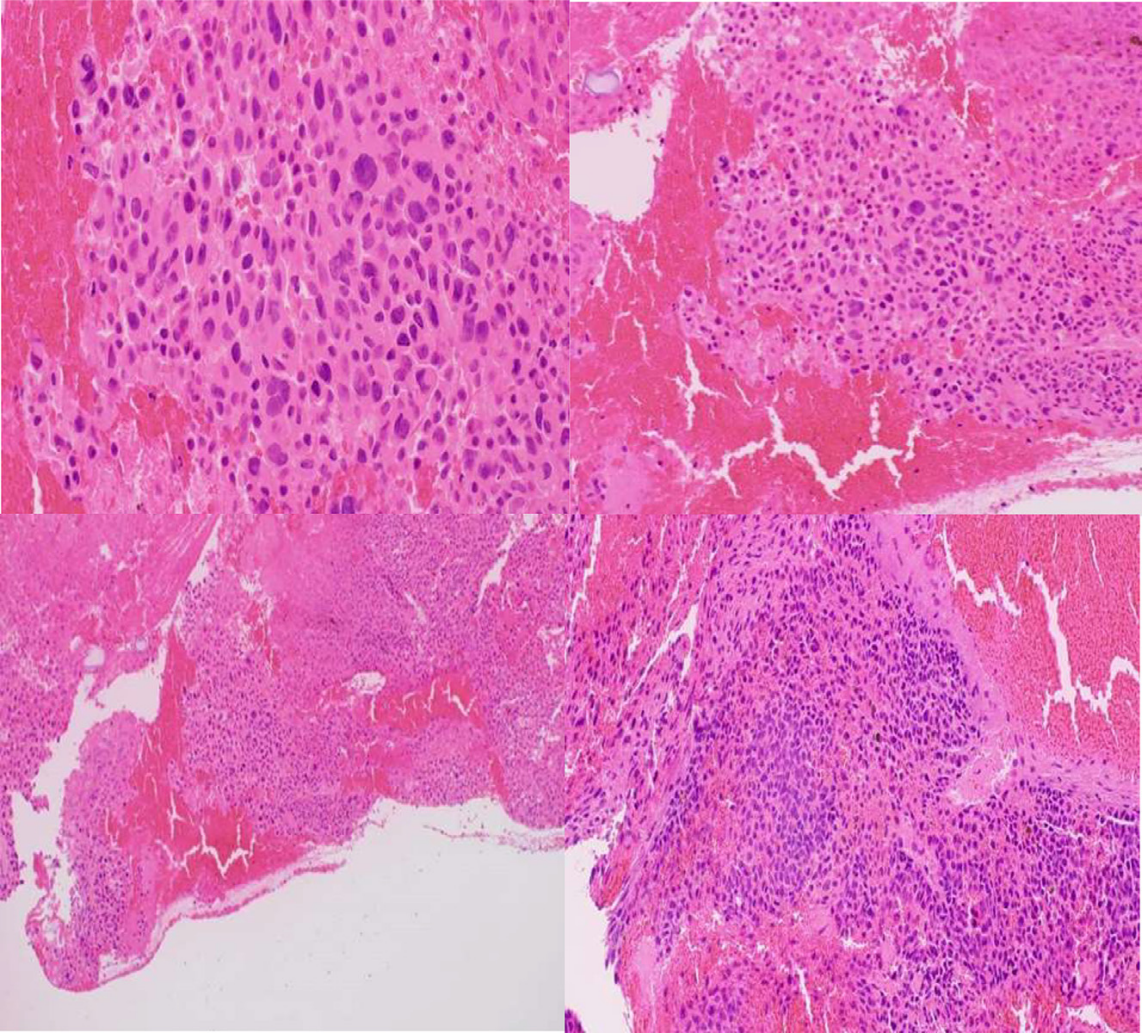
High-grade malignant melanoma;stain used is Hematoxylin and eosin (H&E stain). The section demonstrates solid sheets of neoplastic cells exhibiting nuclear atypia and pleomorphism, with prominent mitoses, necrosis, and hemorrhage. Occasionally, plasmacytoid forms are noted.

Pathology confirmed a high-grade malignant tumor. A biopsy sample sent to the Mayo Clinic corroborated the findings, showing strong positivity for Melan A and HMB45, and negativity for GFAP, EMA, synaptophysin, chromogranin, AE1/AE3, BRAF V600E mutation, and BAP1. It was positive for an NRAS mutation, with a Ki67 proliferation index of up to 20% in certain areas, along with extensive hemorrhage and necrosis, supporting a melanoma diagnosis Fig. 5.

**Fig. 6:**
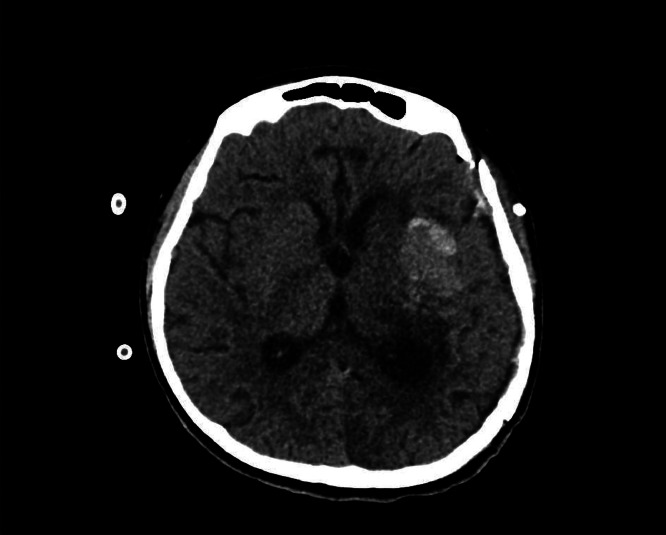
Acute left basal ganglia hemorrhage with perifocal edema and mass effect. This non-contrast axial CT scan of the brain demonstrates an acute intracerebral hemorrhage (ICH). A well-defined hyperdense lesion is present in the left basal ganglia region, consistent with acute blood. Surrounding the hematoma, there is hypodensity suggestive of perifocal edema. Mild mass effect is evident, as indicated by the effacement of the adjacent sulci and compression of the right lateral ventricle. No significant midline shift is observed at this level.

**Fig. 7:**
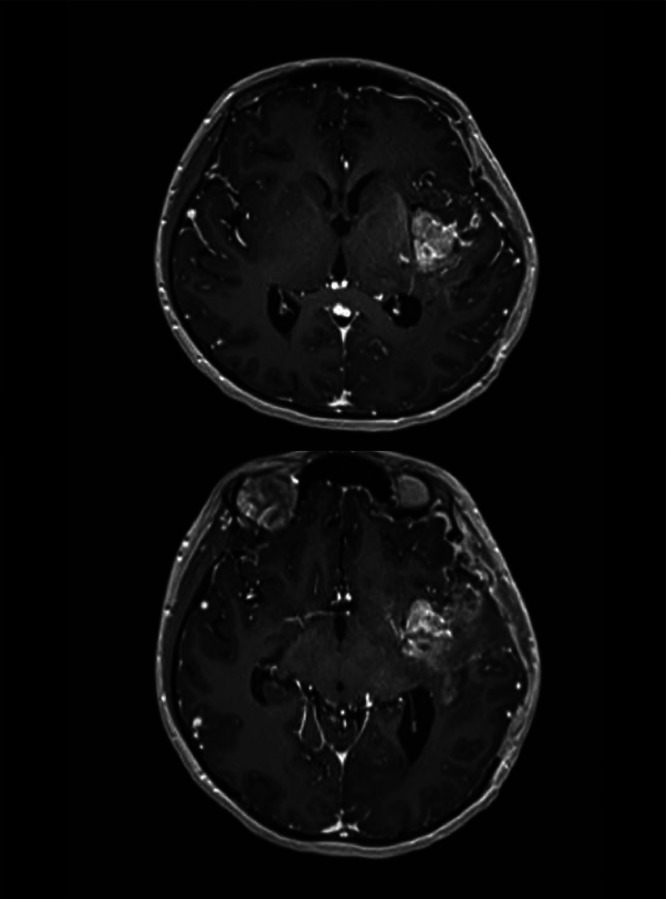
The first MRI image postoperative. The MRI shows a left frontotemporal lesion that appears hyperintense on T1 fat-saturated and T2-weighted images, with diffusion restriction suggestive of a solid lesion. After gadolinium contrast enhancement, heterogeneous enhancement of the lesion is observed, which suggestive of a probable aggressive pathology.

**Fig. 8:**
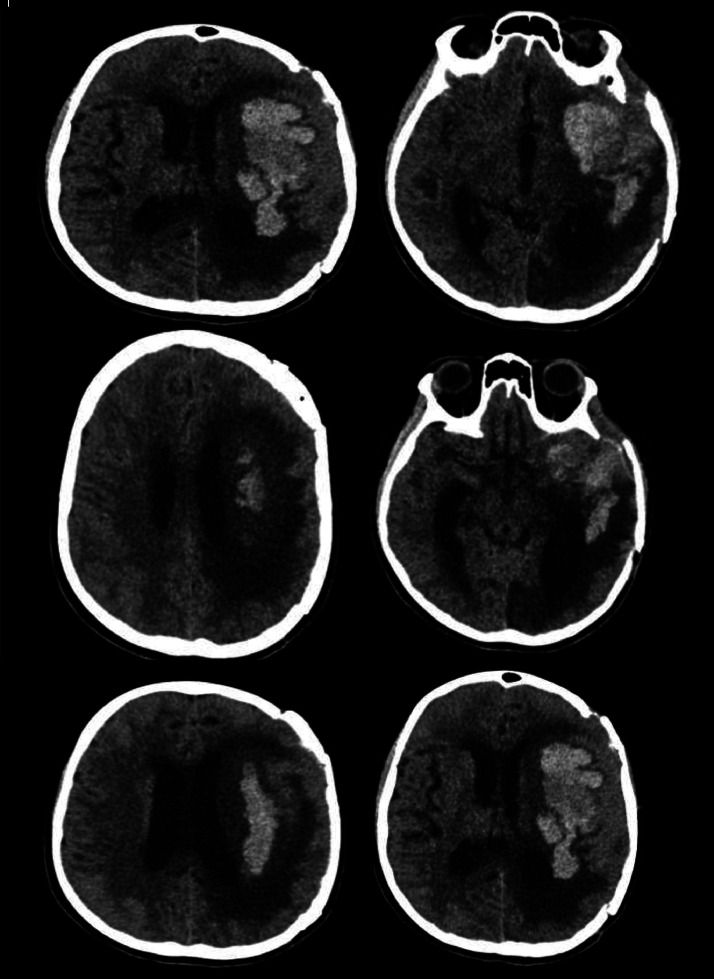
Non-contrast axial CT scan postcraniostomy for the third time. The non-contrast axial CT scan of the brain reveals a large, heterogeneous, hyperdense hemorrhage in the left parietal lobe, with surrounding hypodense vasogenic edema, exerting significant mass effect and causing a midline shift with ventricular compression. The findings are highly suggestive of an acute intracerebral hemorrhage. Possible intraventricular extension is noted, raising concern for hydrocephalus.

**Fig. 9:**
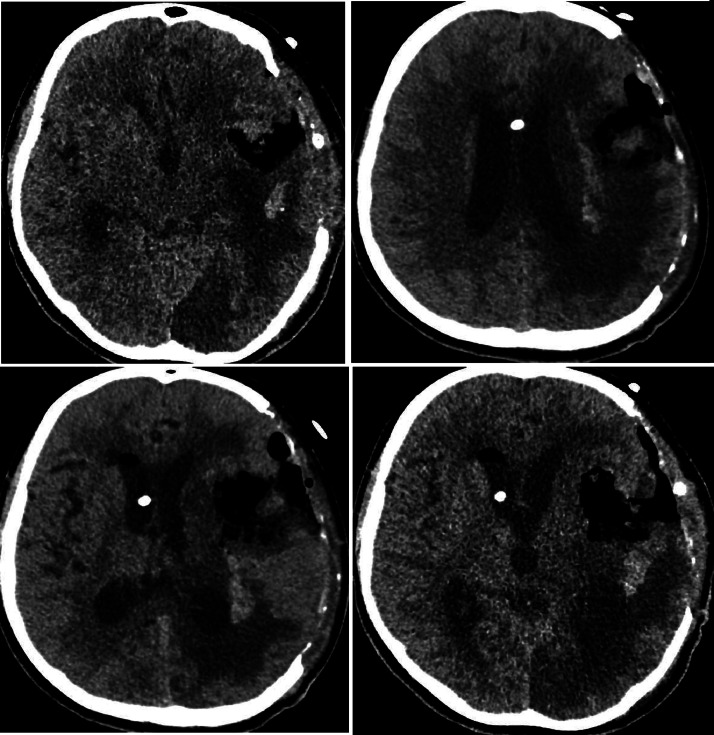
Non-contrast CT brain: Postsurgical changes with mass effect and midline shift. The non-contrast CT brain shows a large left frontoparietal hypodense lesion with significant perifocal edema, causing mass effect, compression of the right lateral ventricle, and a midline shift to the left, indicative of increased intracranial pressure. Postsurgical changes, including a craniotomy defect, are evident, with hyperdense foci along the surgical bed possibly representing hemorrhagic components or postoperative changes. No acute hydrocephalus, basal cistern effacement, or acute intracranial hemorrhage is observed. The findings suggest postsurgical changes with possible residual or recurrent pathology, warranting further MRI evaluation and clinical correlation.

**Fig. 10:**
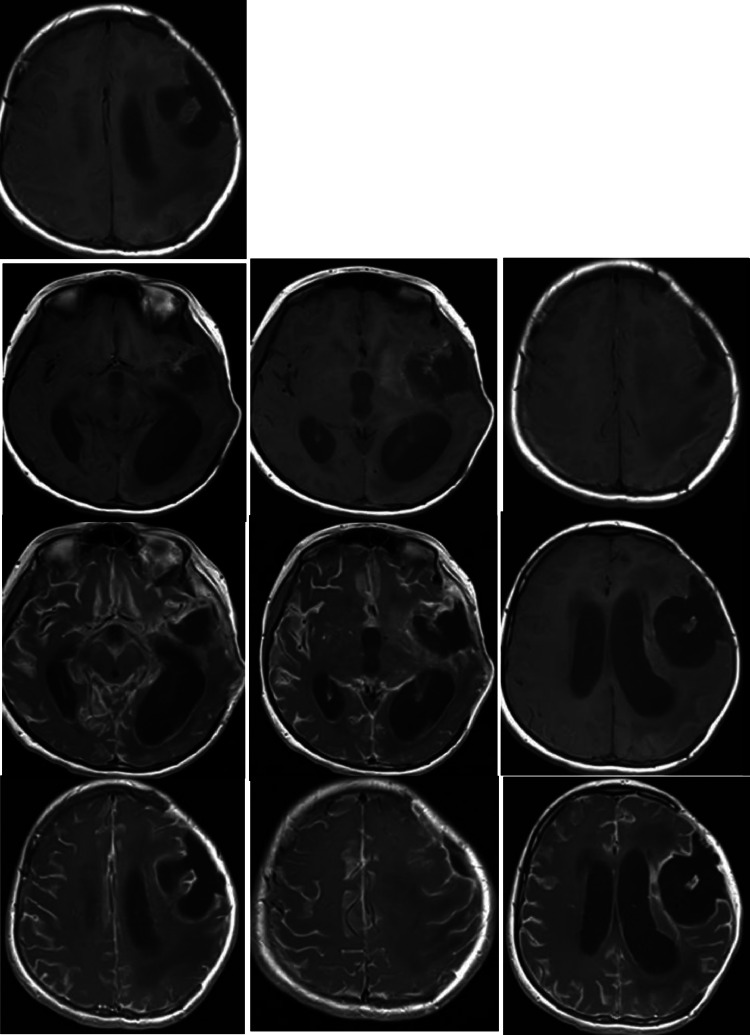
Post-contrast MRI brain: Heterogeneously enhancing left frontoparietal mass. The postcontrast T1-weighted MRI of the brain reveals an excisional cavity with marginal enhancement. There is also diffuse hydrocephalus.

Ophthalmologic and dermatology consultations found no evidence of melanoma elsewhere hence, she was diagnosed with pCNS in the left frontotemporal region. She was started on Nivolumab 1 mg/kg IV over 30 minutes and Ipilimumab 3 mg/kg IV over 1.5 hours for 2 days, followed by Nivolumab 3 mg/kg IV for 30 minutes on the third day. Subsequently, she developed a sudden headache, nausea, vomiting, and dizziness. A non-contrast head CT scan revealed significantly increased hydrocephalus without evidence of recent intracranial bleeding Fig. 6. Under general anaesthesia, she underwent frontotemporal craniotomy for hematoma evacuation follow-up images Fig. 7, Fig. 8, Fig. 9, Fig. 10.

**Fig. 11:**
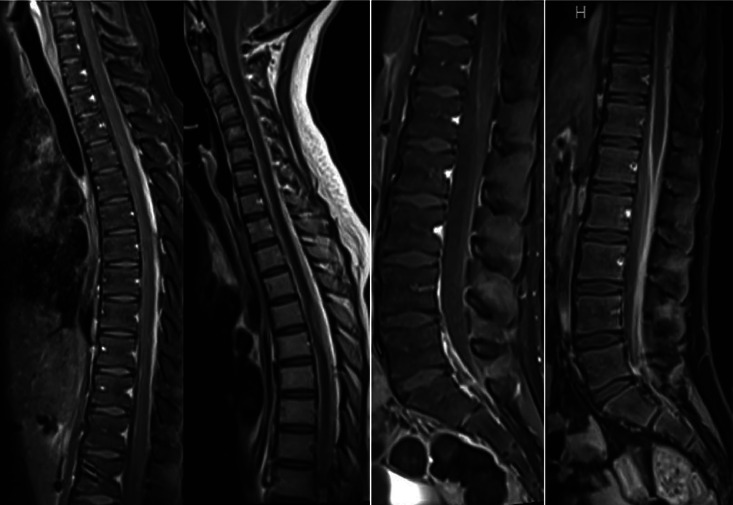
MRI, using T1-weighted and T2-weighted sequences in the sagittal plane of the cervical, thoracic and lumber spine shows the progression of the disease within 3 months (September-November). Significant progression of previously shown leptomeningeal metastases posteriorly (image on the left) and more to the left side extending from T4 down to T10 vertebral body (image on the right). There is an invasion of the posterior lateral aspect of the spinal cord on the left side with more than 50% of the cord involvement at the levels of T5 and T6, with the largest localized lesion measuring 1 × 0.9 cm in axial diameter and extending for 4.2 cm in cranial-caudal dimension compared to 0.6 × 0.7 × 3.6 cm on prior examination. There are also increased leptomeningeal metastases in the cervical, lower thoracic, and lumbar nerve roots, as well as the thecal sac, compared to prior examination. No aggressive bony lesions were seen. There is an increased degree of invasion of the spinal cord mainly on the left side extending from T4 down to T10.

**Fig. 12:**
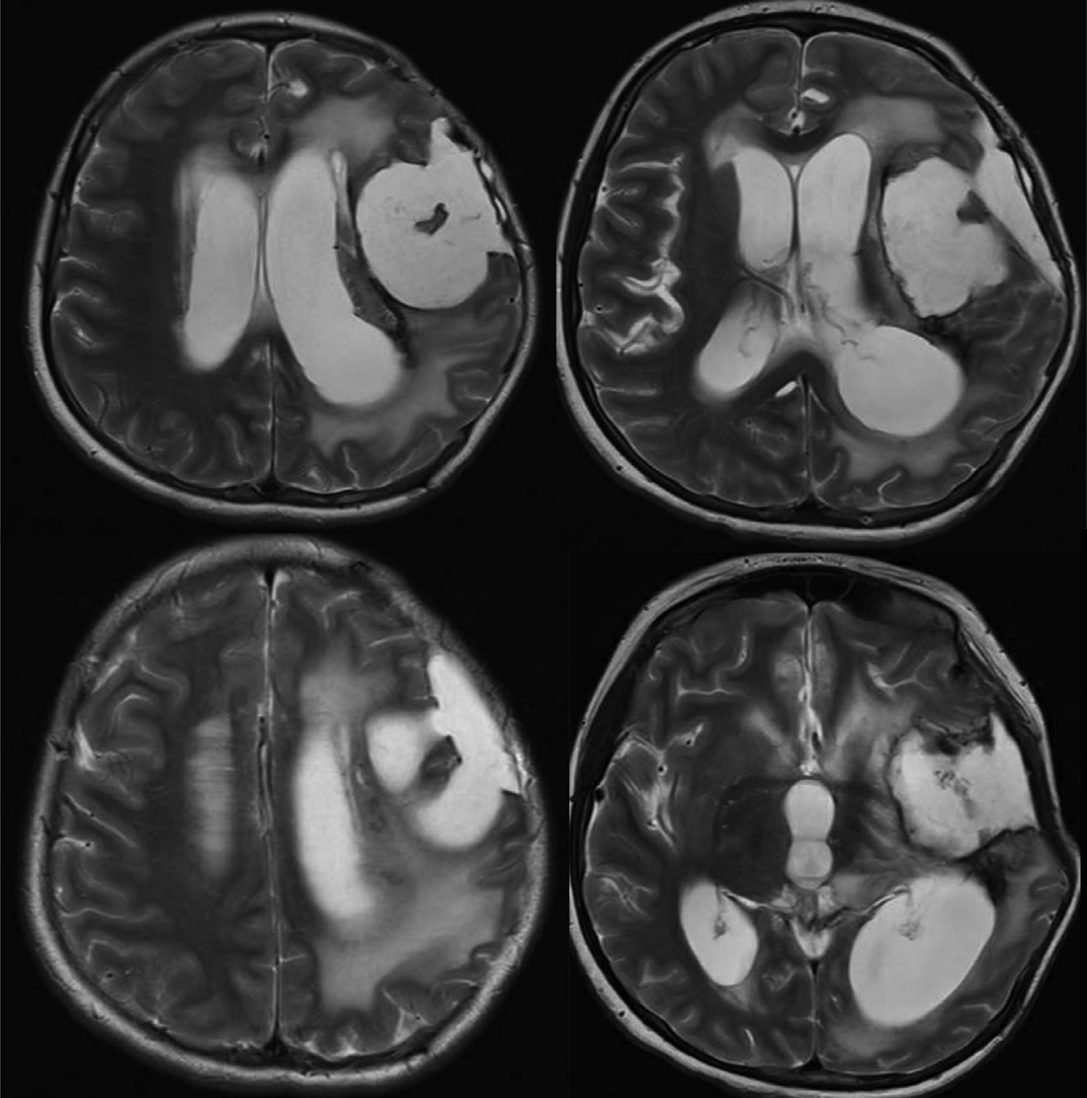
MRI brain (T2-weighted): Large left frontoparietal cystic lesion with mass effect and obstructive hydrocephalus. MRI of the brain (T2-weighted axial images) reveals a large, well-defined, hyperintense cystic lesion in the left frontoparietal region, causing significant mass effect with compression of the adjacent lateral ventricle and a mild midline shift. Associated vasogenic edema extends into the surrounding white matter, and moderate ventricular dilation suggests obstructive hydrocephalus. Postsurgical changes, including a craniotomy defect, are noted without evidence of acute hemorrhage.

**Fig. 13:**
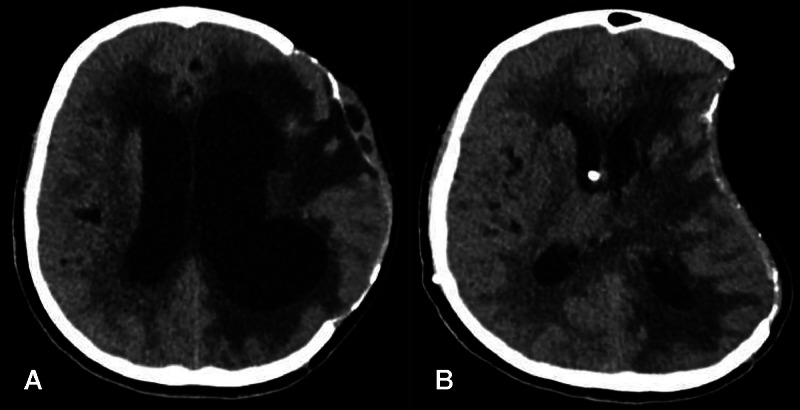
Per and post VP shunt. (A) Non-contrast CT scan of the brain. Due to increased intracranial pressure, an urgent decompressive craniectomy was performed, and a VP shunt was inserted. In (A), the left hemisphere is seen herniating through the craniectomy site. (B) Shows a reduction in pressure after VP shunt insertion." (B) Status post-VP shunt CT scan of the brain. A previous left frontotemporal-parietal craniotomy is noted, along with associated postsurgical changes. A new right transfrontal drain is present, with its tip located within the right lateral ventricle. Small foci of intracranial air are observed, consistent with recent intervention. There has been an improvement in hydrocephalus, with the bifrontal lateral ventricle now measuring up to 51 mm (previously 59 mm).

**Fig. 14:**
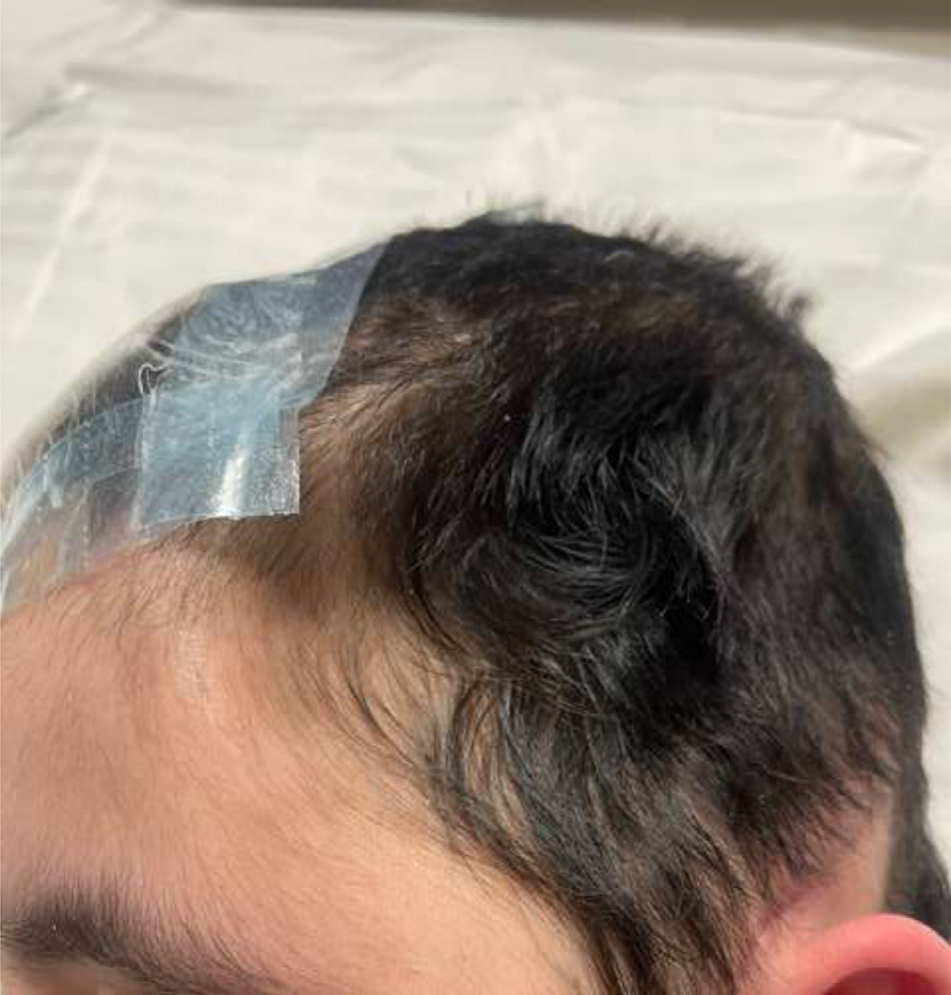
The close-up image shows the head of a young girl after undergoing multiple craniotomy/cranioplasty. The patient's head demonstrates postsurgical changes consistent with prior cranioplasty/craniostomy. The surgical site appears well-healed with no signs of infection, erythema, or dehiscence. Mild scalp edema is present, but no fluctuance or abnormal fluid collection is observed.

She was monitored in the pediatric intensive care unit for 3 days, with intracranial pressure controlled using an external ventricular drain adjusted between 12 and 15 mmHg, required a Ventriculoperitoneal shunt (VP). A magnetic resonance imaging (MRI) of the brain and spine revealed thoracic leptomeningeal metastasis from T1 to T11 Fig. 11, Fig. 12. A postoperative VP shunt placement resulted in slight improvements on a subsequent non-contrast CT scan Fig. 13. The patient's postoperative head image is shown in Fig. 14.

**Fig. 15:**
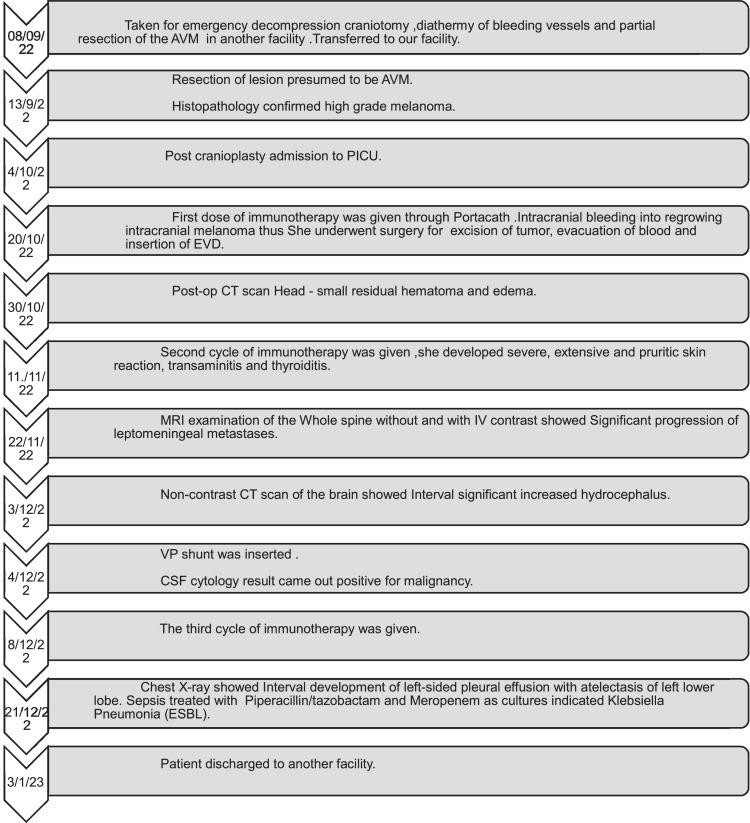
Timeline of the most important events. The full sequence of events of the patient since addition till discharge.

Due to financial constraints, she was transferred to another hospital for continued care. Despite timely interventions, she died due to leptomeningeal disease and elevated intracranial pressure shortly after her transfer. The timeline of the patient most important events is displyed in Fig. 15.

## Discussion

pCNS melanoma is an exceedingly rare condition worldwide, with most reported cases originating from countries like Japan, the Eastern United States, and Europe [2]. In a review of 19 cases between 1989 and 2015, Suranagi et al. [6] identified a male predominance, contrasting with our case. Quillo-Olvera et al. reported the lobes (53.1%), especially the frontal lobe, followed by the posterior fossa (17.3%) and the pineal region (13.6%), as the most common sites for primary brain melanoma [7,8]. Our patient's left frontotemporal tumor aligns with this distribution, but the rarity of such presentations lies in the fact that this tumor is seen in pediatric patients, with only a few cases reported [9,10].

One of the rarest forms of pCNS melanoma, primary intracranial leptomeningeal melanomatosis, presents with diffuse tumor infiltration into the subarachnoid space [11]. Although this was not the case with our patient, she demonstrated other hallmark features of pCNS melanoma, including intracranial hypertension and hydrocephalus. Her symptoms of chronic headaches, nausea, and vomiting, combined with imaging findings, pointed to increased intracranial pressure—a common but challenging complication. Unlike other cases with prominent gait disturbances, our patient's course was dominated by rapid neurological decline due to progressive hydrocephalus.

The radiological findings of pCNS melanoma on a head CT scan often show hyperdense lesions that enhance with the administration of contrast material due to the melanin content within the tumor cells.

On MRI, these tumors often exhibit hyperintensity on T1-weighted images and iso- to hypointensity on T2-weighted images. The hyperintensity on T1-weighted images is a hallmark feature due to the paramagnetic properties of melanin, including Schwannomas [12], while the iso- to hypointensity on T2-weighted images is less specific but still indicative of melanin content [[13], [14], [15]]. In glioblastoma, the pattern is reversed, showing iso- to hypointense on T1-weighted images and hyperintense on T2-weighted images [16]. It is important to note the radiographic differences between pCNS melanoma and AVMs, especially since a non-contrast CT brain scan can be misleading at first. Therefore, digital subtraction angiography and MRI are the best imaging modalities.

On MRI, AVMs typically show flow voids on T2-weighted images and may exhibit susceptibility changes, indicating blood deoxygenation [17,18].

Additionally, pCNS melanoma may show diffuse marked dural and leptomeningeal contrast enhancement on MRI, which can be indicative of leptomeningeal spread or invasion [19,20]. This was the case in our patient..

In addition to imaging, pathology plays a crucial role as a diagnostic tool, with markers like Melan A and HMB-45, along with the NRAS mutation, being the predominant markers identified in primary central nervous system melanoma.

It is noteworthy that mutations in GNAQ and GNA11 are prevalent in pCNS melanocytic tumors, with GNAQ mutations identified at a higher percentage compared to the latter [21,22].

Fewer mutations have been noted to exhibit aggressive behavior, such as the BAP1 loss mutation [21,22].

The role of NRAS mutations, especially in codon 61, is crucial in the development and progression of pCNS melanoma, alongside pathological markers like Melan A and HMB-45. These mutations cause the activation of subsequent pathways that promote uncontrolled proliferation of cells. This mutation accounted for the early presentation in our case and the aggressive tumor behavior, characterized by rapid proliferation and poor prognosis [[23], [24], [25], [26]].

Interestingly, the longest surviving PIMM case involved a male patient with primary pineal malignant melanoma, who remained recurrence-free for 18 years [27]. Studies emphasize the importance of confirming a tumor's primary nature via thorough systemic evaluations, including FDG-PET and biopsy, to differentiate it from metastatic melanoma [28]. This was rigorously adhered to in our patient's workup, which confirmed pCNS melanoma.

Surgical intervention remains a cornerstone of management, often coupled with postoperative radiotherapy. However, our patient was treated with an adjuvant immunotherapy regimen comprising Nivolumab 1 mg/kg IV and Ipilimumab 3 mg/kg IV [29]. While this approach aligns with emerging therapeutic trends, her rapid disease progression, likely compounded by leptomeningeal spread, highlights the limitations of current treatment modalities in pediatric cases.

The possible risk factors may help direct healthcare workers to patients at higher risk. One such factor that may allow for early detection is the presence of congenital melanocytic nevi, and neurocutaneous melanosis these individuals mostly have NRAS mutations, as it is frequently observed within this population. A greater overall genetic predisposition is a crucial factor to consider during history taking [[24], [25], [26], [30], [31]].

For families of patients with cancers that have a genetic predisposition, it is recommended to refer them to a preventive cancer clinic for further screening.

## Conclusion

Our patient's case underlines the unique challenges of diagnosing and managing pCNS melanoma in children. Despite surgical resection and innovative immunotherapy, her disease course was marked by rapid progression, culminating in her tragic death. This case highlights the critical importance of early diagnosis, multidisciplinary care, and continued research into more effective therapies for this rare and aggressive condition. Understanding the nuances of such cases can inform future strategies to improve outcomes for patients with pCNS melanoma.

## Patient consent

Written informed consent was obtained from the patient for publication of this case report and accompanying images. A copy of the written consent is available for review by the Editor-in-Chief of this journal on request.
